# The prevalence of endoscopic gastric mucosal damage in patients with rheumatoid arthritis

**DOI:** 10.1371/journal.pone.0200023

**Published:** 2018-07-09

**Authors:** Saki Tsujimoto, Sho Mokuda, Kenichiro Matoba, Akihiro Yamada, Kazuo Jouyama, Yosuke Murata, Yoshio Ozaki, Tomoki Ito, Shosaku Nomura, Yasuaki Okuda

**Affiliations:** 1 Department of Internal Medicine, Center for Rheumatic Diseases, Dohgo Spa Hospital, Matsuyama, Ehime, Japan; 2 First Department of Internal Medicine, Kansai Medical University, Hirakata, Osaka, Japan; 3 Department of Clinical Immunology and Rheumatology, Hiroshima University Hospital, Hiroshima, Hiroshima, Japan; Sapienza University of Rome, S. Andrea Hopital, ITALY

## Abstract

**Objectives:**

Rheumatoid arthritis (RA) patients often take non-steroidal anti-inflammatory drugs (NSAIDs) and corticosteroids as supportive drugs. In this study, we investigated the prevalence of endoscopic gastric damage and their prescribed medications under an actual clinical condition.

**Methods:**

We collected the data of 1704 RA patients who underwent upper gastrointestinal fiberscopy. Gastric mucosal erosion and ulcer were classified using modified LANZA score. We analyzed these data with a multiple regression analysis.

**Results:**

The prevalence of endoscopic gastric mucosal damage in these RA patients was 16.7% (285 cases). A multiple regression analysis indicated that prednisolone (PSL), NSAIDs and proton pump inhibitors (PPIs) were independent risk factors associated with the modified LANZA score. PSL and NSAIDs were positively correlated with the score, while the administration of PPIs was inversely correlated with the score. The modified LANZA score in RA patients treated with both PSL and NSAIDs was significantly higher than that in those treated with PSL alone (no NSAIDs use).

**Conclusions:**

Our findings suggest that PSL and NSAIDs were exacerbating factors for gastric mucosal damage, while PPIs usage was a protective factor. And, the combined usage of corticosteroids and NSAIDs may induce the development of gastric ulcers.

## Introduction

Rheumatoid arthritis (RA) is a systemic autoimmune disease, characterized by synovitis with bone erosion and joint cartilage degradation [1]. Several decades ago, the primary target of treatment for RA was pain relief. Over the years, however, many progressive drugs able to modify the prognosis of RA have been developed, such as conventional synthetic biological disease-modifying antirheumatic drugs (csDMARDs), including methotrexate (MTX) and biological DMARDs (bDMARDs) [2,3]. These drugs have the potential to induce remission in RA patients [2,3]. However, pain control drugs, represented by non-steroidal anti-inflammatory drugs (NSAIDs) and corticosteroids, are still needed for RA patients who suffer from control failure and progressive joint destruction. A major side effect of these supportive drugs is gastroduodenal mucosal disorder, including erosions, ulcers, bleeding and perforation [4–6].

Cyclooxygenase (COX) catalyzes the first committed step in the synthesis of prostaglandin (PG), which is derived from membrane phospholipids. Two COX isoenzymes are known: COX-1 and COX-2 [7,8]. COX-1 is expressed constitutively by most cells, including the gastrointestinal tract, and mediates the production of PGs related with gastric protection [7,8]. On the other hand, the expression of COX-2 is induced by inflammatory cytokines, growth factors and injury. COX-2-mediated PGs, mainly PGE_2_, can exacerbate invasive pain by collaborating with bradykinin [7–10].

NSAIDs inhibit the activity of COX and regulate inflammation and its pain through the inhibition of PG biosynthesis. The dual inhibition of COX-1 and COX-2 leads to the suppression of inflammatory reactions and gastric mucosal disorder simultaneously. Some authors have reported that the prevalence of gastric and duodenal ulcers in chronic arthritis patients treated with NSAIDs is 5 to 15 times the expected prevalence in a healthy population [11]. Patients at high risk for hemorrhaging and perforation from NSAIDs-induced gastroduodenal ulcers should be considered for prophylaxis with proton pump inhibitors (PPIs), H2 receptor antagonists (H2RAs) and misoprostol [12–17]. Some clinical trials have shown that COX-2-selective NSAIDs are as effective at inducing analgesia as non-selective NSAIDs (COX-1 and COX-2 inhibitors) while reducing the risk of peptic ulceration [9,10].

Corticosteroids also inhibit production of PGs through the regulation of COX-2 expression. Corticosteroids stimulate gastric acid secretion and decrease gastric mucus via the suppression of PGs in the gastric mucosa [18]. Whether or not single treatment with corticosteroids can induce gastric ulcers is controversial [19]. Meanwhile, some authors have reported that patients treated with NSAIDs had an estimated relative risk associated with current corticosteroid use of 4.4 (CI, 2.0–9.7). Therefore, the combined use of corticosteroids and NSAIDs can lead to gastric ulcer [20].

Most of RA patients still require corticosteroids and NSAIDs for pain management, therefore they might be a high risk for developing gastric erosion and ulcer. Nevertheless, epidemiological investigations of the gastrointestinal damage in RA patients under actual clinical conditions has seldom been reported, although the prevalence of NSAIDs-related gastrointestinal damage mainly in osteoarthritis (OA) has been described and some controlled trial in RA patients has been reported [4,6]. In our present study, we examined 1704 RA patients who underwent upper gastrointestinal fiberscopy to determine the prevalence of endoscopic gastric damage and analyzed the relationship between their endoscopic findings and prescribed medications.

## Materials and methods

### Study design and subjects

This cross-sectional prospective study was approved by the clinical ethics committees of Dohgo Spa Hospital (approval number H29-009). We collected the subjects undergone upper gastrointestinal fiberscopy from January 2008 through December 2015 at Dohgo Spa Hospital. Subjects’ written informed consents were obtained before performing fiberscopy. They met the 1987 revised classification criteria for RA [21]. The reasons of this examination were preoperative evaluation and screening for RA complications such as reactive AA amyloidosis in most cases. The exclusion criteria were as follow: follow-up fiberscopy within 6 months, history of gastrectomy, history of combination therapy with both PPIs and H2RAs.

### Collection of clinical data

We extracted the following data from medical records: age, sex, disease duration of RA, Steinbrocker radiological stage and functional class scores. We also evaluated the patients’ history of taking medications related to gastroduodenal mucosal damage. These medications were NSAIDs, including celecoxib (400 mg per day), etodolac (400 mg per day), meloxicam (10 mg per day), indomethacin (50 mg per day), loxoprofen (180 mg per day), diclofenac (75 mg per day), sulindac (300 mg per day), zaltoprofen (240 mg per day), nabumetone (800 mg per day), ampiroxicam (27 mg per day) and lornoxicam (12 mg per day). Required drugs were not included. Celecoxib, etodolac and meloxicam are also COX-2-selective NSAIDs; PPIs, including lansoprazole, omeprazole, rabeprazole and esomeprazole; H2RAs, including famotidine and ranitidine; gastroprotective drugs, including azulene, teprenone, rebamipide, misoprostol, sucralfate, ecabet, polaprezinc, plaunotol, gefarnate, irsogladine and dicyclomine; and oral corticosteroids (calculated as milligrams of prednisolone per day).

### Detection of *Helicobacter pylori* infection

The definition of *Helicobacter pylori (H*. *pylori)* infection required positive results on one or more of the following tests: a histological examination, rapid urease test, serum anti-*H*. *pylori* IgG antibody and *H*. *pylori* antigen in stool. Biopsy specimens were obtained from the antrum and lower body. The same samples were used for the histological examination evaluated by Giemsa staining and/or the rapid urease test (CLO test, Halyard Health, Inc.). Anti-*H*. *pylori* IgG antibody in the serum was measured with an enzyme immunoassay (EIA) (E-Plate Eiken H pylori antibody II; Eiken Chemical Co., Ltd.,). *H*. *pylori* antigen in the stool was measured with an enzyme-linked immunosorbent assay (ELISA) (Meridian Bioscience). *H*. *pylori* infections in patients treated with PPIs were detected by methods other than the rapid urease test.

### Findings of upper gastrointestinal fiberscopy and scores

**Fig 1:**
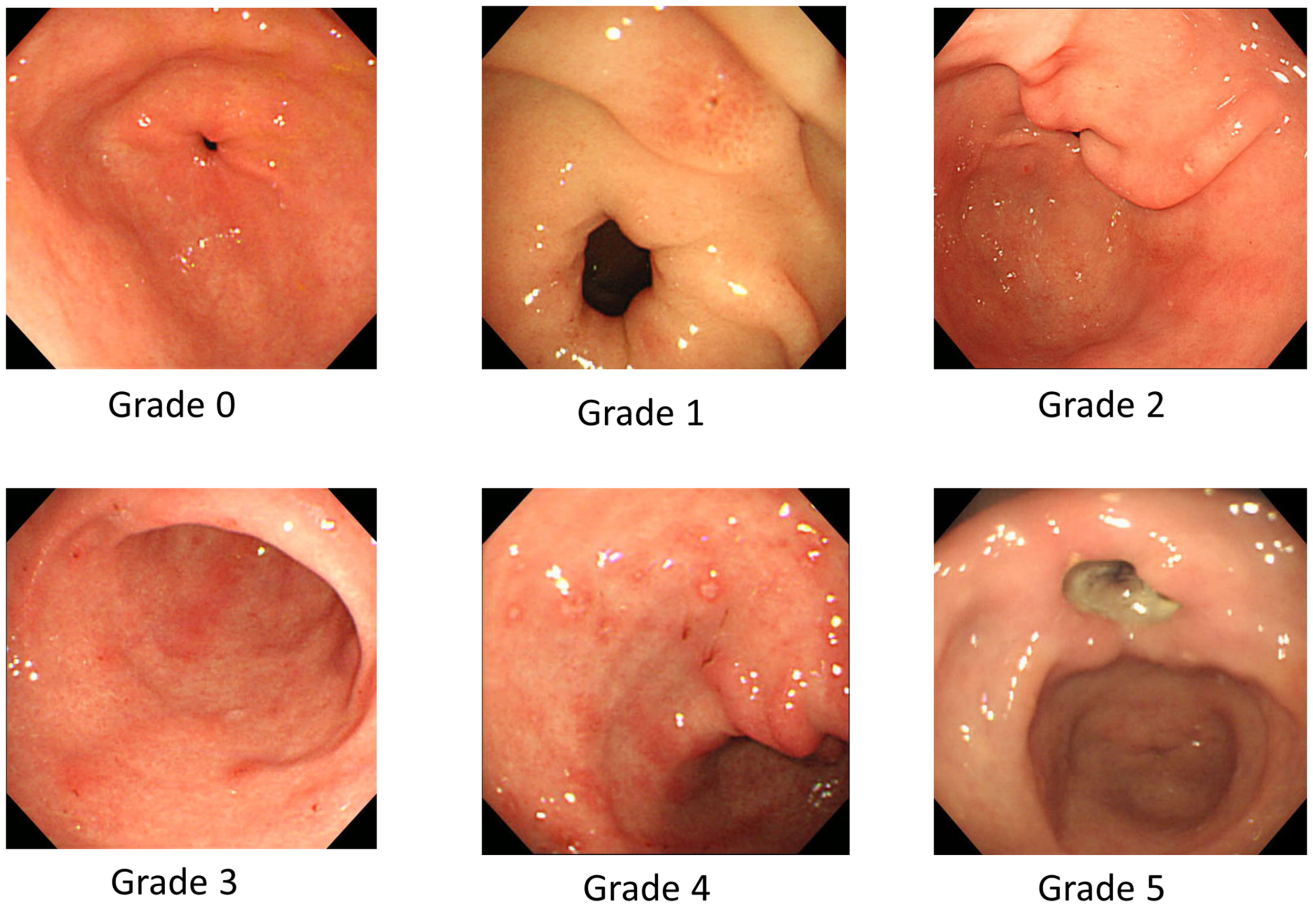
Representative images for the modified LANZA score. These figures indicate representative images of the modified LANZA score.

We classified the cases into 6 grades (from 0 to 5) based on the modified LANZA score for gastric lesions [22,23] as follows (Fig 1): Grade 0 = No erosions or bleeding sites; Grade 1 = 2 or fewer erosions or bleeding sites localized in 1 area of the stomach; Grade 2 = 3 to 5 erosions or bleeding sites localized in 1 area of the stomach; Grade 3 = Erosions of bleeding sites in 2 areas of the stomach, or at least 6 erosions in 1 area but no one more than 10 in the stomach as a whole; Grade 4 = Erosions or bleeding sites in 2 areas of the stomach, or at least 6 erosions in 1 area but no more than 10 in the stomach as a whole; Grade 5 = Gastric ulcer.

### Statistical analyses

In order to calculate the independent risk factors for the modified LANZA score, we performed single regression analyses using Pearson’s correlation coefficient with significant at the p<0.1 level. An adjusted multiple regression analysis was then carried out. The significant of difference between two groups was determined by Student’s t-test. The significant of difference between four groups was determined by one-way analysis of variance (ANOVA), followed by Tukey-Kramer test. Data processing and analyses were conducted using the Graph Pad Prism 7 software program (GraphPad Software, Inc., La Jolla, CA, USA).

## Results

### Patient characteristics and prevalence of gastric mucosal damage

A total of 1704 RA patients were entered into this study. The clinical characteristics of this study population are shown in Table 1. The majority of these patients were female (87.3%), the mean age was 69.3 years, and the mean disease duration of RA was 18.7 years. Among these patients, 59.2% (1008 cases) were treated with NSAIDs and 76.7% (1307 cases) with corticosteroids. The mean dose of oral corticosteroids was 3.7 mg prednisolone (PSL) per day, and 39.0% (665 cases) were treated with PPIs, 26.1% (445 cases) with H2RAs and 34.7% (592 cases) with gastroprotective drugs. The positive rate of *H*. *pylori infection* was 33.3% (69 cases of 207 cases).

**Table 1:** Baseline characteristics of this study.

Characteristics	Total	With or without gastric mucosal damage	
		With erosion or ulcer	Without erosion
Number	1704	285	1419
Age (years), mean±SD	69.3±11.4	69.6±10.5	69.2±11.6
Female, n (%)	1488(87.3%)	248 (87.0%)	1240 (87.4%)
Disease duration (years), mean±SD	18.7±13.5	19.6±14.2	18.5±13.4
Steinbrocker stage			
- stage I, n (%)	59 (3.5%)	6 (2.1%)	53 (3.7%)
- stage II, n (%)	156 (9.2%)	22 (7.7%)	134 (9.4%)
- stage III, n (%)	492 (28.9%)	97 (34.0%)	395 (27.8%)
- stage IV, n (%)	997 (58.5%)	160 (56.1%)	837 (59.0%)
Steinbrocker class			
- class I, n (%)	21 (1.2%)	2 (0.7%)	19 (1.3%)
- class II, n (%)	82 (148.2%)	121 (42.5%)	700 (49.3%)
- class III, n (%)	699 (41.0%)	132 (46.3%)	567 (40.0%)
- class IV, n (%)	163 (9.6%)	30 (10.5%)	133 (9.4%)
Current use of prednisolone, n (%)	1307 (76.7%)	227 (79.6%)	1080 (76.1%)
PSL dose (oral, prednisolone, mg/day), mean±SD	3.7±3.0	4.2±3.7	3.6±2.9
Duration of prednisolone (years), mean±SD	19.3±13.2	19.1±13.6	19.4±13.1
Current use of NSAIDs, n(%), (a)	1008 (59.2%)	193 (67.7%)	815 (57.4%)
Duration of NSAIDs (years), mean±SD	18.0±13.7	17.3±13.7	17.9±13.7
COX-2-selective/ NSAIDs, n (%), (b)	595 (59.0%)	99 (51.3%)	496 (60.9%)
Current use of PPIs, n (%), (c)	665 (39.0%)	97 (34.0%)	568 (40.0%)
Current use of H2RAs, n (%), (d)	445 (26.1%)	80 (28.1%)	365 (25.7%)
Current use of gastroprotective drugs, n (%), (e)	592 (34.7%)	109 (38.2%)	483 (34.0%)
Modified LANZA score, mean±SD	0.56±1.42	3.37±1.63	-
Positive rate of *H*. *pylori* infection, n (%), (f)	69 / 207 (33.3%)	19 / 65 (29.2%)	50 / 142 (35.2%)

^(**a**)^ celecoxib, etodolac, meloxicam, indomethacin, loxoprofen, diclofenac, sulindac, zaltoprofen, nabumetone, ampiroxicam, lornoxicam;

^(**b**)^ celecoxib, etodolac, meloxicam;

^(**c**)^ lansoprazole, omeprazole, rabeprazole, esomeprazole;

^(**d**)^ famotidine, ranitidine;

^(**e**)^ azulene, teprenone, rebamipide, misoprostol, sucralfate, ecabet, polaprezinc, plaunotol, gefarnate, irsogladine, dicyclomine.

^(**f**)^ A total of 207 cases of 1704 cases were tested.

PSL, prednisolone; NSAIDs, non-steroidal anti-inflammatory drugs; COX, cyclooxygenase; PPIs, proton pump inhibitors; H2RAs, histamine H 2-receptor antagonists.

The prevalence and severity of gastric mucosal damage in these RA patients were as follows: 83.3% (1419 cases) of grade 0; 3.2% (55 cases) of grade 1; 3.2% (54 cases) of grade 2; 1.8% (30 cases) of grade 3; 1.3% (22 cases) of grade 4 and 7.3% (124 cases) of grade 5. In our population, 16.7% (285 cases) had some degree of endoscopic gastric mucosal damage.

### Risk factors for gastric erosion/ulcer in RA patients

We then performed regression analyses to determine the independent risk factors for gastric mucosal damage, as classified using the modified LANZA score. First, we performed single regression analyses. The results showed that there were significant differences between the modified LANZA score and the following variables: age, Steinbrocker functional class, PSL dose, use of NSAIDs, PPIs and gastroprotective drugs (p<0.1) (Table 2). We then carried out a multiple regression analysis for these sorted variables, with results indicating that the Steinbrocker functional class, PSL dose, use of NSAIDs and PPIs were independent risk factors associated with the modified LANZA score (p<0.05) (Table 3). The Steinbrocker functional class, PSL and NSAIDs were positively correlated with the modified LANZA score, so we took these to be exacerbation factors against gastric mucosal damage. In contrast, PPIs usage was inversely correlated with the modified LANZA score, suggesting it was a protective factor.

**Table 2:** Univariate analyses for comparisons between the modified LANZA score and each independent variable.

Variable	Coefficient of determination (r^2)	p-value
Age	0.00178	0.082*
Sex	0.0000122	0.886
Steinbrocker stage	0.000372	0.427
Steinbrocker class	0.00498	0.036**
Disease duration	0.000397	0.413
PSL dose	0.00512	<0.001***
Duration of PSL	0.000129	0.680
Usage of NSAIDs	0.00875	<0.001***
Duration of NSAIDs	0.0000699	0.791
PPIs	0.00194	0.070*
H2RAs	0.0000719	0.726
Gastroprotective drugs	0.00228	0.049**
*H*. *pylori* (a)	0.00331	0.410

PSL, prednisolone; NSAIDs, non-steroidal anti-inflammatory drugs; PPIs, proton pump inhibitors; H2RAs, histamine H2-receptor antagonists.

^(**a**)^ A total of 207 cases of 1704 cases were tested.

Data were analyzed using Pearson’s correlation coefficient,

*p<0.1,

**p<0.05,

***p<0.01.

**Table 3:** A multivariate analysis to determine the relationship between the modified LANZA score and independent risk factors.

Variable	Regression coefficient	Standard error	p-value	95%CI (lower limit)	95%CI (upper limit)
Constant	-0.553	0.278	0.0471	-1.098	-0.00712
Age	0.00515	0.00330	0.119	-0.00133	0.0116
Steinbrocker class	0.129	0.0556	0.0202*	0.0202	0.238
PSL dose	0.0381	0.0115	<0.001**	0.0156	0.0606
Usage of NSAIDs	0.279	0.0702	<0.001**	0.141	0.417
PPIs	-0.208	0.0731	0.00455**	-0.351	-0.0643
Gastroprotective drugs	0.102	0.0726	0.159	-0.0402	0.244

PSL, prednisolone; NSAIDs, non-steroidal anti-inflammatory drugs; PPIs, proton pump inhibitors. Data were analyzed using a multivariate analysis,

*p<0.05,

**p<0.01.

**Fig 2:**
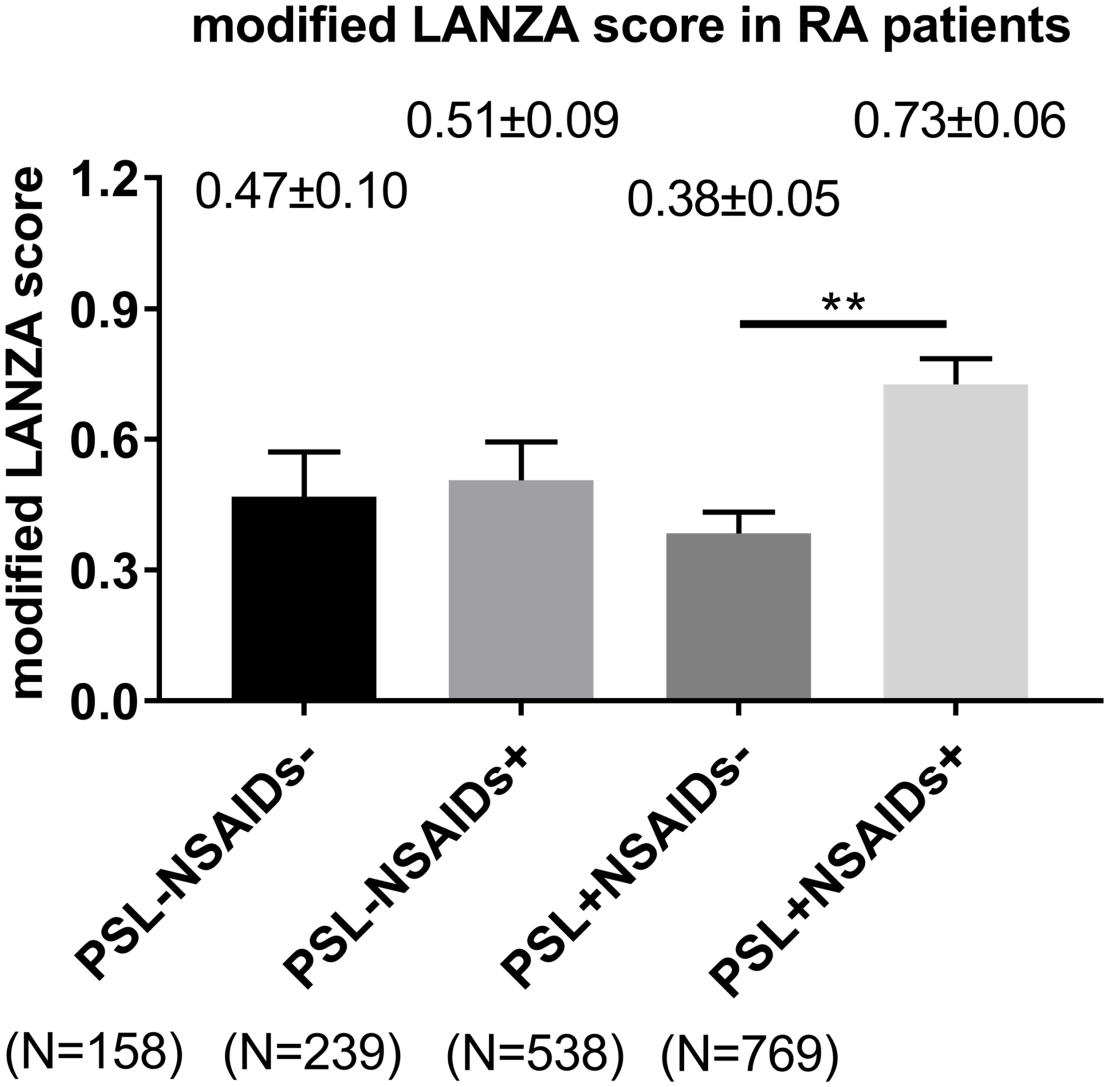
A comparison of the modified LANZA score based on the NSAIDs and PSL treatment status. Values are shown as the mean±SEM (standard error of the mean). Data were analyzed using Tukey-Kramer test, **p<0.01. PSL, prednisolone; NSAIDs, non-steroidal anti-inflammatory drugs.

Further analyses showed that there was difference in the modified LANZA score based on the NSAIDs treatment status among RA patients treated with PSL. The score of a group of neither NSAIDs nor PSL use (n = 158), a group of NSAIDs alone (no PSL use) (n = 239), a group of PSL alone (no NSAIDs use) (n = 538) and a group of treatment with both NSAIDs and PSL (n = 769) were average ± standard error of mean (SEM) 0.47±0.10, 0.51±0.089, 0.38±0.049 and 0.73±0.058, respectively. Among these four groups, the modified LANZA score in RA patients treated with both NSAIDs and PSL was significantly higher than that in those treated with PSL alone (no NSAIDs use), analyzed using Tukey-Kramer test (p<0.01; Fig 2).

### Protective effect of COX-2-selective NSAIDs in RA patients

**Fig 3:**
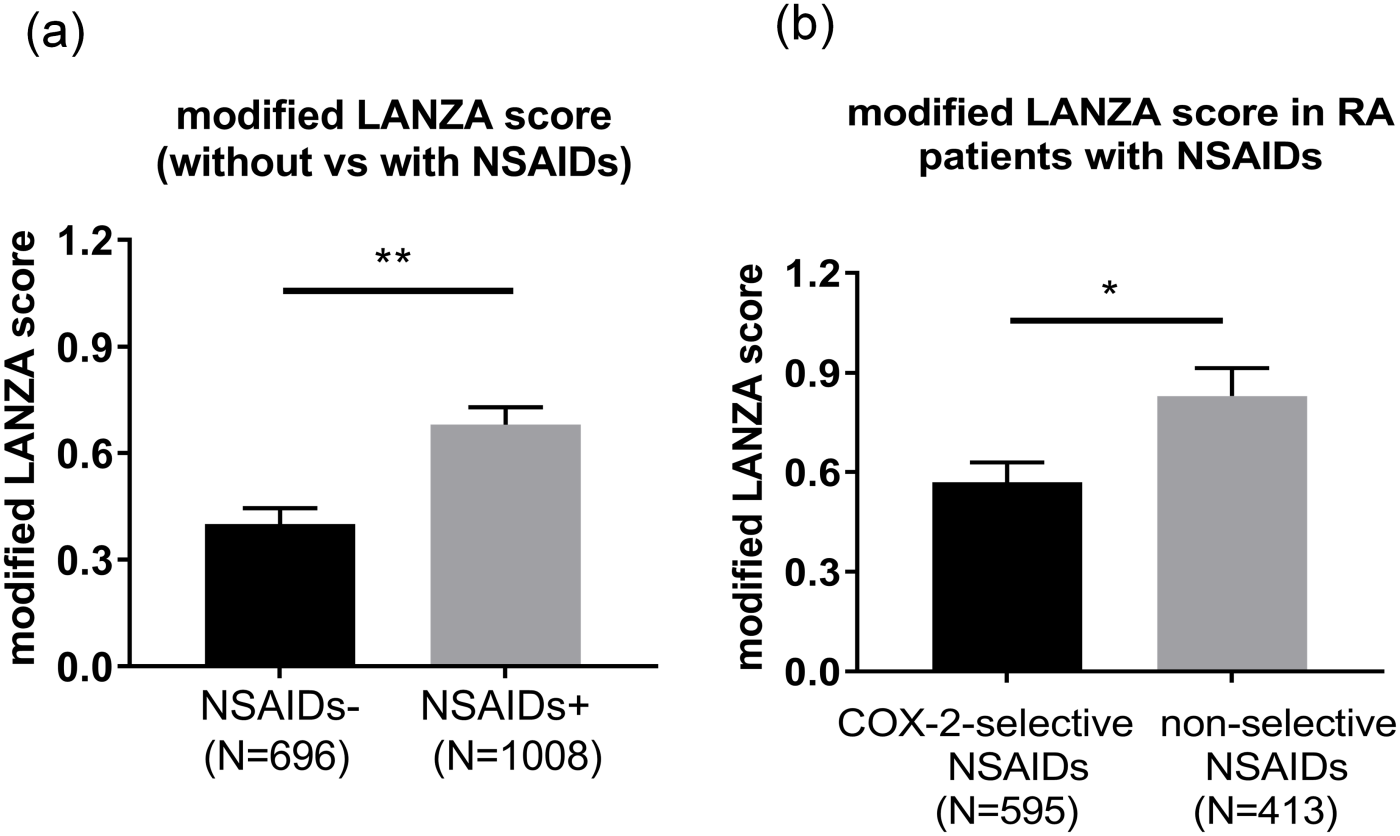
A comparison of the modified LANZA scores between those treated with COX-2-selective inhibitors and those treated with non-selective COX-1 and COX-2 inhibitors. (a) A comparison of the modified LANZA scores between patients treated with and without NSAIDs. (b) A comparison of the modified LANZA score between patients treated with COX-2-selective inhibitors and non-selective COX-1 and COX-2 inhibitors. Value are shown as the mean±SEM. Data were analyzed using a t-test, *p<0.05, **p<0.01. NSAIDs, non-steroidal anti-inflammatory drugs; COX, cyclooxygenase. Celecoxib, etodolac and meloxicam are COX-2-selective NSAIDs.

In our regression analyses, the variable of NSAIDs included all types of NSAIDs. As described above, COX-2-selective NSAIDs are reported to reduce the risk of peptic ulceration compared to non-selective NSAIDs (COX-1 and COX-2 inhibitors) [9,10]. Therefore, we also investigated the modified LANZA score of COX-2-selective NSAIDs in RA patients. The score in RA patients treated with NSAIDs (n = 1008) (average±SEM 0.68±0.049) were higher than in those not treated with NSAIDs (n = 696) (0.40±0.045) (p<0.001) (Fig 3a). We then divided the NSAIDs-treated patients into 2 groups: those treated with COX-2-selective NSAIDs (n = 595) and those treated with non-selective NSAIDs (n = 413). The modified LANZA scores of the RA patients treated with COX-2-selective NSAIDs were significantly lower than the scores of those treated with non-selective NSAIDs (0.57±0.060 and 0.83±0.084, respectively; p = 0.010; Fig 3b).

## Discussion

Although RA patients who are taking these drugs are at high risk of gastroduodenal injury, few epidemiological reports have described the relationship between RA and gastroduodenal mucosal damage under actual clinical conditions. [24]. In our present study, we analyzed 1704 RA patients and their endoscopic gastric mucosal findings (modified LANZA score) to clarify the relationship between treatment and gastric mucosal ulcer. Our data showed that 16.7% of total RA patients and 19.1% of RA patients treated with NSAIDs had gastric mucosal damage (data not shown). This prevalence is about the same as in the previous reports for patients with arthritis [4–6].

In addition, our multiple regression analysis identified the Steinbrocker functional class and usage of PSL, NSAIDs and PPIs as independent risk factors associated with the modified LANZA score. Specially, PPIs usage was inversely correlated with the modified LANZA score, while the Steinbrocker functional class and use of PSL and NSAIDs were positively correlated with the modified LANZA score. These data suggest that PPIs can help prevent gastric mucosal damage. By contrast, H2RAs usage was not an independent risk factor. It has already been reported that PPIs and high-dose H2RAs have prophylaxis effects on NSAIDs-associated gastric ulcer [12,13]. However, in Japan, only low-dose H2RAs is covered by the medical insurance system, limiting the use of other dosage. For example, some guidelines for the prophylaxis of NSAID-associated gastric ulcer recommended 40 mg of famotidine twice a day [25], while the Japanese medical insurance system limits the use to ≤20 mg twice a day. This dosage limitation might have caused H2RAs usage not to be included as an independent risk factor.

In the present study, the Steinbrocker functional class and use of PSL and NSAIDs were positively correlated with the modified LANZA score, indicating that these factors exacerbated gastric mucosal damage. Piper et al. reported that the estimated relative risk of gastric ulcer among current users of oral corticosteroids was 2.0 (95% confidence interval [CI] 1.3–2.0). Interestingly, the estimated relative risk for corticosteroid users treated without NSAIDs was 1.1 (95%CI 0.5–2.1), while the risk for those treated with NSAIDs was 4.4 (95% CI 2.0–9.7) [20]. This trend in non-RA patients was similar to that observed in RA patients in our present study. Therefore, the concomitant treatment of corticosteroids and NSAIDs was deemed likely to increase the risk of gastric ulcer in RA patients. And, treatment with COX-2-selective NSAIDs might help resolve this issue in RA patients. It has already been reported that COX-2-selective NSAIDs have a gastric ulcer-protective effect compared with non-selective NSAIDs in RA patients [26,27]. Our present study also demonstrated that the modified LANZA score of RA patients treated with COX-2-selective NSAIDs was lower than that in RA patients treated with non-selective NSAIDs. These data implied that the treatment with COX-2-selective NSAIDs might reduce the gastric mucosal damage in RA patients.

To our knowledge, the relationship between the Steinbrocker functional class and LANZA score has never been reported before. Some authors have reported that an advanced age is a risk factor for NSAIDs-related gastric ulcer [11,28]. In the present study, most of the subjects were elderly, and over 50% were in functional classes III and IV. Although age was not included as an independent risk factor, age can affect the functional class. RA patients with an impaired activity of daily living might be at high risk for developing gastric mucosal damage.

*While H*. *pylori* infection tends to be associated with gastric ulcers, this is unlikely to be the case in RA patients or NSAIDs-treated subjects. Ishikawa et al. reported that the prevalence of gastroduodenal lesions did not seem to depend upon *H*. *pylori* infection in RA patients [29]. Matsukawa et al. found no evidence that *H*.*pylori* infection increases gastric ulcer formation in NSAIDs users [30]. We similarly detected no relationship between *H*. *pylori* infection and gastric ulcer.

In summary, our present findings suggest that clinicians should carefully monitor RA patients treated with both corticosteroids and NSAIDs and consider treatment with PPIs and COX-2-selective NSAIDs.

Limitations. We did not analyze the prevalence of duodenal erosion or ulcer in this study because the prevalence of endoscopic duodenal damage was found to be extremely low (0.8%) in another preliminary study conducted by our group. Most of our subjects were elderly, so further studies in youth and middle aged-patients are needed. Furthermore, as this was a cross-sectional study, case or randomized controlled trials are needed to identify the causality between the risk factors and the LANZA score.

## Supporting information

S1 Datafile Data set for regression analysis. (XLSX)
